# A 23-Year-Old Female with a Mixed Germ Cell Tumor of the Pituitary Infundibulum: The Challenge of Differentiating Neoplasm from Lymphocytic Infundibuloneurohypophysitis—A Case Report and Literature Review

**DOI:** 10.1155/2014/129471

**Published:** 2014-06-18

**Authors:** Sann Yu Mon, Hussain Mahmud, Munira Abbasi, Geoff Murdoch, Juan C. Fernandez-Miranda, Paul A. Gardner, Sue M. Challinor

**Affiliations:** ^1^Division of Endocrinology and Metabolism, University of Pittsburgh Medical Center, 200 Lothrob Street, BST 1140, Pittsburgh, PA 15213, USA; ^2^Division of Endocrinology and Metabolism, University of Pittsburgh Medical Center, 3601 Fifth Avenue, Suite 3B, Pittsburgh, PA 15213, USA; ^3^Division of Neuropathology, UPMC Presbyterian Hospital, M8723 South Tower, 200 Lothrop Street, Pittsburgh, PA 15213, USA; ^4^University of Pittsburgh Medical Center, UPMC Presbyterian Hospital, Suite B-400, 200 Lothrop Street, Pittsburgh, PA 15213, USA; ^5^Division of Endocrinology and Metabolism, University of Pittsburgh School of Medicine, Falk Medical Building, Suite 580, 3601 Fifth Avenue, Pittsburgh, PA 15213, USA

## Abstract

The pathologic spectrum of diseases that infiltrate the pituitary infundibulum includes a broad variety of clinical entities. There are significant differences in the prevalence of these etiologies depending on the age of presentation. Lymphocytic infundibuloneurohypophysitis (LINH) predominates over other causes of infundibular disease in adults over age 21. Differentiating LINH from other causes of infundibular disease can be difficult because the various etiologies often have similar clinical presentations and radiologic imaging characteristics. We report the first case in an adult of a mixed germ cell tumor comprised of germinoma and embryonal cell carcinoma infiltrating the pituitary infundibulum. In our case, a 23-year-old female was initially misdiagnosed as having LINH. She presented with panhypopituitarism and diabetes insipidus, which is the most common initial presentation in both entities. The two diagnoses are difficult to distinguish based on MRI imaging, CSF findings, and histopathological examination. Our case demonstrates the need for close follow-up of patients with isolated lesions of the pituitary infundibulum and reinforces the need for biopsy of an infundibular lesion when progression of disease is demonstrated. In our case, biopsy with comprehensive immunohistochemical staining was the sole means of making a definitive diagnosis.

## 1. Introduction

The pathologic spectrum of diseases that infiltrate the pituitary infundibulum includes a broad variety of clinical entities. Congenital, developmental, inflammatory, infectious, granulomatous, neoplastic, and traumatic etiologies have all been described in the literature [1]. However, there are significant differences in the prevalence of these etiologies depending on the age of presentation [2]. Lymphocytic infundibuloneurohypophysitis (LINH) predominates over other causes of infundibular disease in adults over age 21, accounting for 26% of all cases. Neurosarcoidosis and metastatic lesions are also among the more common causes of pituitary infundibular disease in adults and account for 14% and 11% of all cases, respectively. Lymphoma and leukemia infiltrating the infundibulum together comprise another 10% of adult cases. In children, the more common infundibular lesions include pituitary hypoplasia in 60% and Langerhans histiocytosis in 19% [2]. Suprasellar germ cell tumors (GCT) are also more common in children than adults, representing 10% versus 5% of all infundibular lesions, respectively [2]. GCTs can be histologically subcategorized into the more common germinomas and less prevalent nongerminomatous germ cell tumors. Infiltration of the pituitary infundibulum by a primary mixed germ cell tumor (MGCT), a combination of two or more GCT subtypes, is exceedingly rare; to our knowledge, only 5 cases have been reported in adults [3–8].

Differentiating LINH from other causes of infundibular disease can be difficult because the various etiologies often have similar clinical presentations and radiologic imaging characteristics. In many cases, biopsy is the only way to make the definitive diagnosis, but carries the risk of causing panhypopituitarism (PHP), and is therefore usually deferred in a patient with intact pituitary hormone function. A thorough search for the presence of other systemic involvement with disease and examination of cerebrospinal fluid may provide clues for the diagnosis. In light of the high prevalence of LINH, adult patients with isolated lesions of the pituitary infundibulum are often followed conservatively, since LINH generally has a self-limited course and treatment is unlikely to reverse pituitary hormone deficiencies. Close follow-up of these patients is important in order to assess any progression of disease via imaging or visual field determination. Progressive growth of the lesion on serial MR imaging, worsening of visual field deficits, and/or progressive loss of pituitary hormonal function should raise concern for a neoplasm. In that case, and if the diagnosis is still not apparent, biopsy of the infundibular lesion should be considered in order to guide appropriate management [1–9].

Herein, we report the first case in an adult of a MGCT comprised of germinoma and embryonal cell carcinoma infiltrating the pituitary infundibulum. In our case, a 23-year-old female was initially misdiagnosed as having LINH. She presented with PHP and diabetes insipidus (DI), which is the most common initial presentation in both entities. In addition, the two diagnoses are difficult to distinguish based on MRI imaging, CSF findings, and histopathological examination. Our case demonstrates the need for close follow-up of patients with isolated lesions of the pituitary infundibulum and reinforces the need for biopsy of an infundibular lesion when progression of disease is demonstrated. In our case, biopsy with comprehensive immunohistochemical staining was the sole means of making a definitive diagnosis.

## 2. Case Report

**Figure 1:**
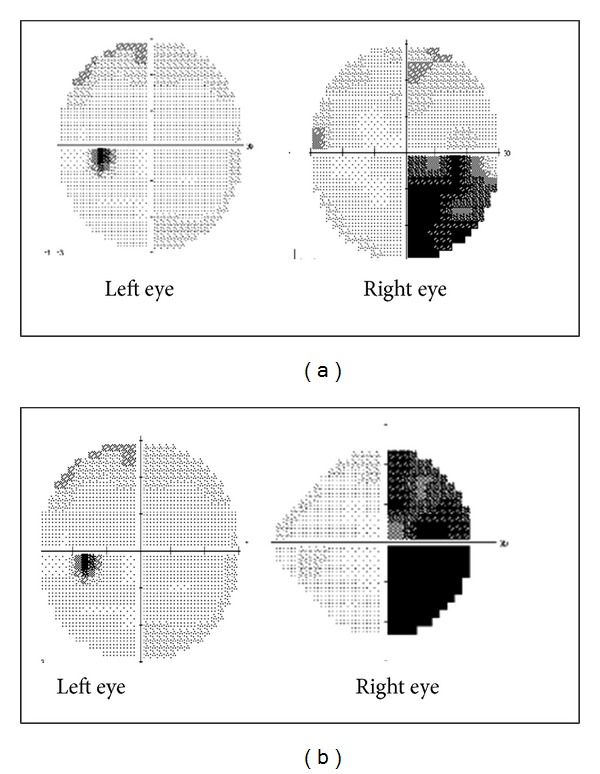
Comparison between initial visual field testing and 3-month follow-up visual field testing.** (**a) Initial visual field test showing right eye inferotemporal visual field deficit. (b) Worsening of visual field deficit in right eye at 3-month follow-up visual field testing.

**Figure 2:**
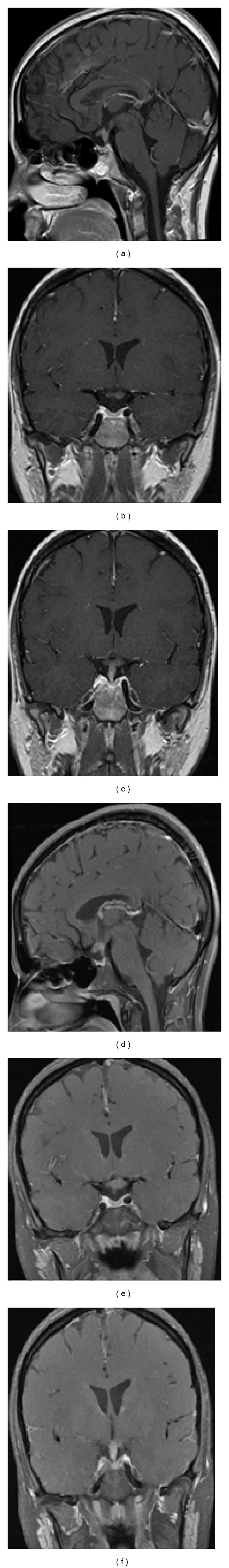
Comparison of MRI pituitary with and without contrast images between initial evaluation and follow-up evaluation. At the initial evaluation ((a) Sagittal slice; (b) and (c) Coronal slices), the pituitary infundibulum thickening was seen with minimal involvement of optic chiasm. At the follow-up evaluation 3 months later ((d) Sagittal slice; (e) and (f) Coronal Slices), abnormal enlargement and enhancement of infundibulum worsened with progressive involvement of left aspect of optic chiasm and proximal portion of left optic nerve.

A 23-year-old female was referred to our pituitary center for evaluation of a pituitary infundibular lesion. She initially presented a few months earlier, with recurrent, intractable left frontotemporal headaches. Pituitary MRI showed an infiltrative hypothalamic and infundibulum lesion, with atrophy of the pituitary gland. When a detailed history was obtained, she reported amenorrhea for 3 years, progressive fatigue, cold intolerance, dry skin, and weight gain, as well as polyuria and polydipsia of 2-year duration. The initial physical examination revealed short stature (4′′11′′) but was otherwise normal, with well-developed breast and normal pubic hair development for her age. The initial ophthalmological examination was consistent with a right inferotemporal visual field deficit (Figure 1(a)). The basic laboratory testing revealed normal blood counts, blood urea nitrogen, creatinine, and electrolytes. Hormonal analysis demonstrated central adrenal insufficiency, central hypothyroidism, and central hypogonadism (Table 1). The 24-hour urine collection revealed 8 liters of urine output in 24 hours and further testing confirmed the diagnosis of DI. Cerebrospinal fluid (CSF) studies were negative for infection, malignant cells, human chorionic gonadotropin (hCG), A-fetoprotein (AFP), and angiotensin converting enzyme [10] (Table 1). The initial MRI reported abnormal enhancement and 6.3 mm thickening of the infundibulum with involvement of the left aspect of the optic chiasm and proximal portion of the left optic nerve (Figures 2(a)–2(c)).

A variety of different etiologies for her hypothalamic/infundibular lesion were considered, but initially, the diagnosis of LINH was favored, based on her age at presentation. The patient was managed conservatively with close clinical follow-up and physiologic hormone replacement with hydrocortisone, levothyroxine, and desmopressin. Three months after her initial assessment, her visual symptoms worsened, with progression of the visual field deficit in the right eye (Figure 1(b)). Repeat MRI imaging of the pituitary reported progressive infiltration of the left optic nerve (Figures 2(d) and 2(e)). The possibility of a neoplastic process was suspected due to these radiographic changes. The patient subsequently underwent uncomplicated endoscopic endonasal biopsy of the lesion.

**Figure 3:**
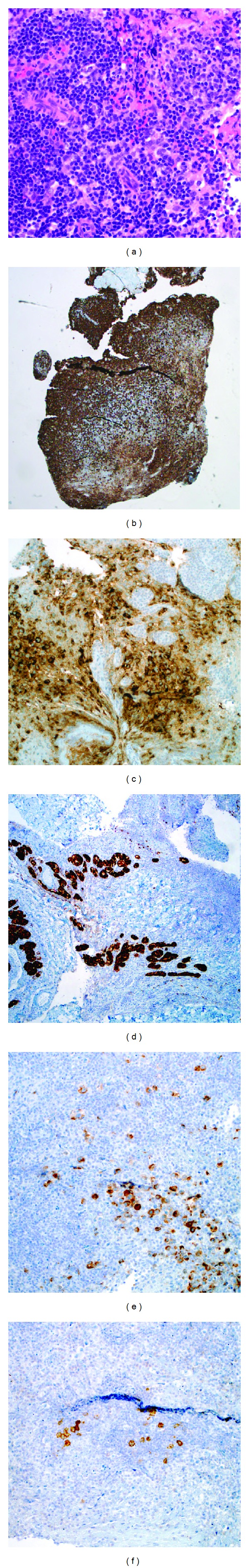
Biopsy of infundibulum: (a) H&E sections of lesion show sheets of small lymphocytes and clusters of larger cells (orig. mag. 20x), (b) CD3 stain of entire biopsy shows predominance of T cells (DAB, orig. mag. 4x), (c) placental alkaline phosphatase (PLAP) immunostain demonstrates that at least some of larger cells are germ cell tumors, (d) keratin immunostain shows nests of epithelial cells consistent pituitary follicles (positive synaptophysin not shown) (DAB orig. mag. 10x), (e) keratin stains also show individual positive cells in area of germ cell tumor, suspicious for an embryonal carcinoma component (DAB, orig. mag. 20x), and (f) CD30 stains confirm presence of embryonal carcinoma component (DAB, orig. mag. 20x).

Surgical pathological slides and immunohistochemical staining results are shown in Figure 3. Although there was lymphocytic predominance, careful examination revealed a scattered group of large atypical cells, which raised the possibility of a neoplasm. The immunohistochemical analysis on paraffin sections was positive for octamer-binding transcription factor (Oct-4), Sal-like protein 4 (SALL 4), and placental alkaline phosphatase (PLAP), which was consistent with germinoma. In addition, a few cells were positive for CD30 and cytokeratin, which suggested the coexistence of embryonal cell carcinoma. Based on the histopathological findings, a diagnosis of MGCT with components of germinoma and embryonal cell carcinoma was confirmed. The patient was subsequently referred to a pediatric neurooncologist who prescribed chemotherapy with five cycles of carboplatinum and etoposide followed by conventional radiotherapy.

## 3. Discussion

Although the overall true incidence of pituitary infundibular lesions remains unknown, these lesions often are discovered incidentally, due to an increase in use of more sensitive and advanced brain imaging studies carried out to investigate nonspecific symptoms or to evaluate hypopituitarism [9]. A retrospective review of the etiologies of pituitary infundibular lesions in patients aged 2 to 82 years concluded that 60 out of 152 (39%) lesions were of unclear etiology, 32% were neoplastic, 20% were inflammatory, and 9% were congenital anomalies. Among the 49 neoplastic lesions, metastatic lesions were the most common (49%) and germinoma was reported in only 6 cases [9].

LINH was suspected in our case initially, because of the higher prevalence of LINH in her age group and the absence of systemic involvement with sarcoidosis or other neoplastic diseases. LINH is of autoimmune origin and is characterized by lymphocytic infiltration in the infundibulum and the neurohypophysis [11]. It was first reported by Saito et al. in 1970 as a cause of idiopathic central DI [12]. The most common presenting symptoms include mass effect symptoms and DI. Other pituitary hormonal deficiencies may present depending on the extent of inflammation. LINH is reported in a wide age range from 3 years to 77 years, with a mean age of 47.3 years [11, 13]. Its prevalence in males and females seems to be similar, with Miyagi et al. reporting a slight male predominance (60%) [14], whereas Takahashi et al. reported 70% female predominance [13]. The classic magnetic resonance imaging findings are diffuse thickening of the pituitary infundibulum and loss of the normal bright spot on T1-weighted images [11]. Although the diagnosis is often made based on clinical presentation and imaging studies, biopsy is the only certain means of diagnosis. The diffuse infiltrate of inflammatory cells in the infundibulum on histological evaluation is the classic diagnostic finding. LINH can be self-limited with spontaneous regression seen in many cases [15].

Primary CNS GCTs are a rare and heterogeneous group of tumors comprised of germinomas and nongerminomatous germ cell tumors. Germinomas are the more common among the primary intracranial GCT, comprising 65% of GCTs, and the majority of germinomas (57%) arise in the suprasellar cistern. Nongerminomatous GCTs include embryonal carcinomas, teratomas, choriocarcinomas, and endodermal sinus tumors. These tumors preferentially involve the pineal gland (68%) and those arising in the suprasellar region are reported in only 4.6% of all intracranial GCTs. The overall prevalence of intracranial GCT is much higher in males; however, suprasellar GCTs are more frequent in females. Germinomas are usually diagnosed between age of 10 and 21 years, whereas nongerminomas are more frequently diagnosed between birth and 9 years [16]. Although intrasellar MGCTs, a combination of two or more subtypes, have been reported occasionally in children, only 5 cases have been described in adults [17–21]. To the best of our knowledge, this is the first case report in an adult of an infundibular MGCT which includes an embryonal carcinoma component.

The clinical triad of DI, pituitary insufficiency, and visual abnormalities is common at presentation with a pituitary GCT, but obstructive hydrocephalus can be seen in the presence of pineal tumors [16]. Radiologically, heterogeneous enhancement of an intrasellar mass with hemorrhagic or cystic components would raise suspicion for a GCT [22]. However, in some cases thickening of the infundibulum is the only radiological finding, especially with early onset small tumors [22]. Although CSF tumor markers such as AFP and hCG may be useful for diagnosing CNS GCT in some patients, the lack of such markers does not preclude the diagnosis of a GCT [23, 24]. In a study of 58 patients with CNS germinomas, 60% were negative for serum and CSF beta hCG [24]. In addition, up to 85–90% of patients with CNS germinomas test negative for serologic hCG and AFP [25]. Therefore, a clinician should be aware that these tumor markers might be well absent in over half of patients with a CNS GCT. Consequently, only a tissue biopsy for histopathologic examination can serve as the basis for a definitive diagnosis. Such a biopsy is, however, frequently delayed due to the risk of pituitary infundibulum disruption and the consequent risk of PHP.

Several case reports have emphasized the similarity in the clinical presentation of LINH and germinomas [3–5, 7, 8]. To the best of our knowledge, 6 adult cases have been reported over the last 15 years where germinoma was misdiagnosed as LINH, based on initial clinical presentation and/or pathologic evaluation (Table 2). In all 6 of these cases, the correct diagnosis was made, subsequently, when the patients were reevaluated for worsening of their clinical conditions [3–8]. Two of these cases required repeat biopsy due to worsening of clinical symptoms and progression of a mass on MRI [4–7]. In these two cases, the initial biopsy reported LINH but was later confirmed to be a germinoma after repeat tissue biopsy with appropriate immunohistochemical staining [4, 7, 8]. In the other 4 cases, LINH was diagnosed based on clinical presentation and a single biopsy later in their course of treatment demonstrated GCT. It is interesting to note that, in our case, the patient had symptoms of hypopituitarism for 2 years prior to presenting with headache, which implies that initially there was slow progression of disease. This indolent pace of disease progression has also been reported in 2 other cases of germinoma (Table 2) that were initially misdiagnosed as LINH. It is difficult to explain the apparent accelerated growth of the lesion over a 3-month period, subsequent to presenting to our clinic (Figures 1 and 2). One potential explanation is that the onset of the more aggressive embryonal cell carcinoma component of her mixed germ cell tumor occurred later in the course of her disease.

There are several reports emphasizing the difficulties encountered in differentiating between LINH and GCT upon histopathologic examination, as lymphocytic infiltration can be seen in both entities. It is not unusual for intrasellar germinomas to be missed, especially when a few scattered neoplastic cells are embedded in diffuse predominant sheets of lymphocytes. Because the lymphocytic infiltration can be so predominant, the identification of the neoplastic GCT may be difficult [26–28]. Proper immunohistochemical staining in these cases is important, not only to identify neoplastic cells, but also to differentiate between the specific subtypes of GCT. The management and prognosis of suprasellar GCT depend on the histologic subtype. While germinomas usually can be treated effectively with radiotherapy alone, various combinations of surgical resection, chemotherapy, and radiotherapy are often required for nongerminomatous GCTs. Germinomas typically have a good prognosis with a 10-year survival rate of 93%, but outcomes for nongerminomatous tumors often are less favorable. Choriocarcinoma and embryonal carcinomas have a poor prognosis with a 3-year survival rate of 27%. The 3-year survival rate can be as low as 9% in some MGCT [29].

## 4. Conclusion

Despite their rare occurrence in adults, clinicians should keep in mind the possibility of a GCT when evaluating patients with an infiltrating disease of the pituitary and infundibulum, even when serum or CSF markers are negative. As our case demonstrates, GCT and LINH can present with highly similar clinical signs and symptoms as well as similar pathological findings on biopsy. In our patient, the invasion of the lesion into the optic chiasm and progression of the pituitary stalk involvement on MRI, along with worsening of the visual field deficits, raised our suspicion for a neoplasm. This finding highlights the need for close follow-up with serial MRI imaging and visual field testing when evaluating these patients. Tissue biopsy with adequate sampling was essential for the correct diagnosis in our case and fortunately was of low risk to the patient, as she presented with PHP, and thus biopsy of the infundibulum would not contribute to further hormonal dysfunction. Comprehensive immunostaining was also important, both to ensure differentiation of GCT from LINH and to determine the subtype of GCT, which guided appropriate treatment.

## Figures and Tables

**Table 1:** Laboratory studies and CSF studies at initial evaluation.

Laboratory tests	Patient's results	Normal/reference range
IGF1	55 ng/mL	182–780 ng/mL
GH	0.10 ng/mL	0.01–8 ng/mL
TSH	2.537 mIU/mL	0.3–5 mIU/mL
Free T4	0.66 ng/dL	0.8–1.8 ng/dL
Total T3	1.08 ng/mL	0.6–1.81 ng/mL
Estradiol	8 pg/mL	Early 10–50; mid 120–375; late cycle 50–155 pg/mL
FSH	1.6 mIU/mL	Follicular 1.4–9.9; midcycle 6.1–17.2; luteal 1.1–9.2 mIU/mL
LH	0.8 mIU/mL	Follicular 1.7–15; midcycle 21.9–56.5; luteal 0.6–16.3 mIU/mL
Prolactin	24.9 ng/mL	0.6–20 ng/mL
ACTH	9 pg/mL	6–50 pg/mL
Cortisol 8 AM	3.7 mcg/dL	4–22 mcg/dL
Alpha subunit	<0.3 ng/mL	<1.5 ng/mL
ACE	40 U/L	9–67 U/L
*CSF *Studies		
Gross Appearance	Clear	Clear
Supernatant	Colorless	Colorless
RBC	1/*μ*L	0/*μ*L
WBC	1/*μ*L	0–5/*μ*L
ACE	<4 ACE units	<4 ACE units
*β* 2 Microglobulin	0.57 mg/L	0.36–2.56 mg/L
Glucose	52 mg/dL	40–85 mg/dL
Protein	26 mg/dL	15–45 mg/dL
Alpha Fetoprotein	<1.0 ng/mL	<1.5 ng/mL
Tumor hCG	1 mIU/mL	Normal

IGF1: insulin like growth factor 1; GH: growth hormone; TSH: thyroid stimulating hormone; FSH: follicular stimulating hormone; LH: luteinizing hormone; ACTH: adrenocorticotropic hormone; CSF: cerebrospinal fluid; RBC: red blood cells; WBC: white blood cells; ACE: angiotensin converting enzymes.

**Table 2:** Six adult cases of germinoma mimicking clinically LINH described in the literature.

Age, sex	Clinical presentation	MRI findings	Positive tumor markers	Immunohistochemical findings	Time course	Treatment
21, M [3]	VFD, PHP, DI	Nodular thickening of stalk	Not done	PLAP CD117	Unclear	Surgery

22, F [4]	VFD, polyuria, weight loss, irregular menstruation	Initial MRI: suprasellar mass with a cystic component MRI 46 months later: mass enlarged invading optic chiasm	Serum and CSF hCG	No report for specific staining	46 months from initial presentation to final diagnosis	CT

24, F [5]	Headache, PHP	Diffuse thickening of stalk	Serum hCG	PLAP	3-week rapid growth of tumor	RT

24, F [6]	PHP, DI	Diffuse thickening of stalk	Negative serum, CSF hCG, AFP	PLAP CD117	Unclear	CT, RT

40, F [7]	Headache, diplopia, PH, DI	Intrasellar mass extending to the suprasellar and the clivus	Serum PLAP	CD43 CD45R0 CD20	4 years from initial presentation to final diagnosis	Surgery

45, M [8]	Headache, VFD, secondary hypogonadism	Intrasellar mass extending into right cavernous sinus	CSF hCG	PLAP	1 month of rapid progression	CT, RT

VFD: visual field defect; PHP: panhypopituitarism; DI: diabetes insipidus; CT: chemotherapy; RT: radiotherapy.
